# Availability of mental health service providers and suicide rates in Slovenia: a nationwide ecological study

**DOI:** 10.3325/cmj.2013.54.444

**Published:** 2013-10

**Authors:** Helena Korošec Jagodič, Tatjana Rokavec, Mark Agius, Peter Pregelj

**Affiliations:** 1 Psychiatric Hospital Vojnik, Vojnik, Slovenia; 2 Institute of Public Health of the Republic of Slovenia, Ljubljana, Slovenia; 3 South Essex Partnership University Foundation Trust, Weller Wing, Bedford Hospital, Bedford, UK; 4 Department of Psychiatry University of Cambridge, Cambridge, UK; 5 Clare College Cambridge, Cambridge, UK; 6 University Psychiatric Hospital Ljubljana, Ljubljana, Slovenia; 7 University of Ljubljana, Faculty of Medicine, Department of Psychiatry, Ljubljana, Slovenia

## Abstract

**Aim:**

To investigate the influence of socioeconomic factors, mental health service availability, and prevalence of mental disorders on regional differences in the suicide rate in Slovenia.

**Methods:**

The effects of different socioeconomic factors, mental health service availability, and mental disorders factors on suicide rates from 2000-2009 were analyzed using a general linear mixed model (GLMM). Pearson correlations were used to explore the direction and magnitude of associations.

**Results:**

Among socioeconomic factors, unemployment rate ranked as the most powerful predictor of suicide and an increase of one unit in the unemployment rate increased regional suicide rate by 2.21 (β = 2.21, 95% confidence intervals [CI] = 1.87-2.54, *P* < 0.001). On the other hand, higher marriage/divorce ratio was negatively related to the suicide rate and an increase of one unit in marriage/divorce ratio reduced regional suicide rate by 1.16 (β = -1.16, 95% CI = -2.20 to -0.13, *P* < 0.031). The most influential mental health service availability parameter was higher psychiatrist availability (4 psychiatrists and more working at outpatient clinics per 100 000 inhabitants), which was negatively correlated with the suicide rate and reduced regional suicide rate by 2.95 (β = -2.95, 95% CI = -4.60 to -1.31, *P* = 0.002). Another negatively correlated factor was the antidepressant/anxiolytic ratio higher than 0.5, which reduced the regional suicide rate by 2.32 (β = -2.32, 95% CI = -3.75 to -0.89, *P* = 0.003). Among mental health disorders, only the prevalence of alcohol use disorders was significantly related to the regional suicide rates and an increase of one unit in the prevalence of alcohol use disorders per 1000 inhabitants increased the regional suicide rate by 0.02 (β = 0.02, 95% CI = 0.01- 0.03, *P* = 0.008).

**Conclusions:**

Besides unemployment, which was a very strong predictor of suicide rates, unequal availability of mental health services and quality of depressive disorder treatment may contribute to variations in suicide rates in different regions.

Suicidal behavior is a significant public health problem and almost 800 000 people throughout the world commit suicide each year (1). Slovenia is a small European country with high suicide rate (2). However, there are big differences in the suicide rate between different regions of Slovenia (3).

Suicidal behavior has multiple causes that are broadly divided into proximal stressors or triggers and predisposition (4). Psychiatric disorder is a major contributing factor and it could be speculated that access to services, which increases the possibility of diagnosis and treatment, could have an impact on regional suicide rates (5). It is also known that on average 55% of suicide victims have no contact with mental health or primary care providers in the month prior to suicide (6). Thus the availability of services may be relevant in explaining geographical variations in suicide incidence (5). Different indicators of mental health availability have been proposed, however it has recently been suggested that the density of mental health professionals and the availability of primary health care providers could be used as core indicators of mental health service quality in different regions (7). It has also been reported that the availability of mental health services may vary substantially between and within countries (8-10).

Suicide rates are higher in rural than in urban regions (11,12), despite higher social support (13), which is considered a protective socioeconomic factor against suicide (14). This could be explained by lower availability of psychiatric services in rural regions (15,16). Furthermore, depression and comorbid alcohol related disorders frequently remain unidentified and thus untreated (17). In the United States, it has been reported that alcohol use disorders had, by far, the lowest appropriate treatment rates of any disorders studied (17). However, studies of the relationship between suicide rates and availability of mental health services are rare (5). Nevertheless, some studies confirm the relationship between the suicide rates and the number of working physicians (18), psychiatrists (19), or outpatient mental health services (20) available.

The aim of this study was to investigate the influence of socioeconomic factors, mental health service availability, and the prevalence of mental disorders on regional differences in suicide rate in Slovenia. The hypothesis was that regions in Slovenia in which there were more mental health care providers would have lower suicide rates than regions in which there were fewer mental health care providers. The models accounted for socioeconomic covariates (average income, unemployment, marriage rate, divorce rate) and mental disorders covariates (yearly prevalence of identified alcohol related disorders/depression disorders/psychotic disorders, prescription rate of antidepressants, and anxiolytics).

## Methods

The Slovene population is divided into 12 different statistical regions: Obalno- kraska, Goriska, Gorenjska, Osrednja-slovenska, Notranjsko-kraska, Jugovzhodna Slovenija, Spodnje posavska, Zasavska, Savinjska, Koroska, Podravska, and Pomurska. The statistical regions of Slovenia are 12 administrative entities created in 2000 for legal and statistical purposes (21).

We obtained information on the suicide rate, population, socio-economic parameters, mental health service availability, prevalence of identified mental disorders, and prescription rate of antidepressants and anxiolytics in each statistical region in each year during a 10- year period (2000-2009). Therefore, there were 120 (12 regions by 10 years) data points. The permission to perform the study was obtained from the Slovenian Medical Ethics Committee.

### Suicide data

The yearly suicide rate (per 100 000 inhabitants) of the total population in the 12 statistical regions was obtained from the Institute of Public Health of the Republic of Slovenia. Since it is mandatory to register all deaths, Slovenia’s population and vital event statistics are accurate and complete. On the other hand, suicides are prone to misclassification during death ascertainment procedures (22). Kapusta et al found out that autopsy rates may affect the validity of suicide mortality statistics (23). For Slovenia, we have no available data regarding the autopsy rate in general. However, in the last study by Zupanc et al conducted in central and northwestern parts of Slovenia, the proportion of suicide victims who were autopsied was 78.1% (from January 1, 2000 through December 31, 2007) (24).

### Population and socio-economic data

The annual population data of the 12 statistical regions were gathered from the National Statistical Office of Slovenia. Common socioeconomic risk factors for suicide including gross domestic product per capita (GDP), unemployment rate, number of marriages (per 1000 population), and number of divorces (per 1000 population) were collected. Unemployment rate is a measure of the prevalence of unemployment and is calculated as a percentage by dividing the number of unemployed individuals by all individuals currently in the labor force.

### Mental health service availability data

Data were obtained from the Institute of Public Health of the Republic of Slovenia. Mental health service availability data including the number of psychiatrists working at outpatients clinic (per 100 000 population), the availability of psychological services (measured as the number of clinical psychologists per 100 000 population), and the availability of primary care doctors (measured as the number of general practitioners (GP) or family physicians (FP) per 100 000 population) were collected.

We calculated the number of psychiatrists on a full- time outpatient equivalent (number of working hours per year divided by 1430 hours as is the standard for Slovenia). We considered only the outpatient psychiatric service without including other psychiatric health service facilities. However, it is the presence of psychiatrists who work with patients in outpatient clinics that is most likely to influence the long term trend in suicide rate, since they, together with primary care doctors, are able to give support and appropriate medication to patients. On the other hand, the role of psychiatrists working in acute wards is to help patients in acute situations. Some authors assessed the impact of psychotherapists’ density on suicide rate (24), but in Slovenia psychotherapy is done mostly by psychiatrists and clinical psychologists. Additionally, we classified psychiatrist availability into higher (≥4 psychiatrists working at outpatient clinics per 100 000 inhabitants) and lower (<4 psychiatrists working at outpatient clinics per 100 000 inhabitants). The number of psychiatrists was arbitrarily set at 4 psychiatrists and more working at an outpatient clinic per 100 000 inhabitants, representing the mental health service availability in Slovenia (25).

### Data on mental disorders

Data were obtained from the Institute of Public Health of the Republic of Slovenia (26). The prevalence of mental disorders (alcohol use disorders, depressive disorders, and psychotic disorders per 1000 population), the number of prescribed anxiolytics and antidepressants per 1000 population, and the antidepressant/anxiolytic ratio (the number of prescribed antidepressants was divided by the number of prescribed anxiolytics) were collected for each year in 12 statistical regions of Slovenia. The antidepressant/anxiolytic ratio was classified as either high (0.5) or low (0.5). The diagnoses in the database were recorded in accordance with the International Statistical Classification of Diseases, 10th revision (ICD-10) (27) (alcohol use disorders, F10.0-F10.9; psychotic disorders F20- F29; depressive disorders F32, F33, and F41.2). Drugs included in the study were classified according to an Anatomical- Therapeutic-Chemical (ATC) drug classification system.

### Statistical analysis

We performed the risk analysis of factors related to suicide rate by examining bivariate associations using the Pearson correlation coefficients. Multivariate analysis of factors significant in bivariate analysis was conducted with a saturated model, with backward deletion excluding the weakest association, until only significant associations remained. For the multivariate analysis, we used general linear mixed model (GLMM). GLMM combines the properties of linear mixed models that incorporate random effects and generalized linear models, which contain data that do not show normal distribution. It is a statistical method for modeling outcome measures as a function of fixed (population) effects, while simultaneously modeling individual subject parameters as random effects, and can accommodate time-dependent covariates as well as missing observations (28). Statistical region was taken as the subject variable and a random effect with socioeconomic factors, mental health service availability, and mental disorders parameters as fixed effects, year as repeating variable, and suicide rate as the dependent variable, and unstructured covariance structure was used. For each parameter that was entered into GLMM as fixed effect, regression coefficient (β) and 95% confidence interval (CI) for regression coefficient were calculated. We used the statistical software SPSS 15.0 (SPSS Inc., Chicago, IL, USA). All reported *P* values were two- tailed, and the level of significance was set at *P* < 0.05.

## Results

The 12 statistical regions cover the entire territory of Slovenia and encompass its entire population (about 2 million 100%). In the northeastern regions (Spodnje posavska, Zasavska, Savinjska, Koroska, Podravska, and Pomurska), mean 10-year suicide rates were high (around 30), in Jugovzhodna Slovenia and Gorenjska they were somewhat lower (around 24), and in the central and southwestern part of Slovenia (Obalno- kraska, Goriska, Osrednje-slovenska, and Notranjsko-kraska) still lower (around 20 and below) (Table 1). Antidepressant/anxiolytic ratio, which represents the prescription practice of antidepressants and anxiolytics, ranged from 0.24 to 1.04 (Table 2).

**Table 1:** Basic demographic data and suicide rate for 12 Slovenian statistical regions for the period from 2000 to 2009. Data are shown as mean.

Statistical regions	Territory (km^2^)	Population (per km^2^)	Male sex (%)	65 y and over (%)	Mean suicide rate
Koroska	1041	70.8	49.9	13.8	33.2
Pomurska	1337	91.9	48.5	15.5	29.8
Podravska	2170	147.5	48.9	15.4	30.1
Savinjska	2384	107.8	49.3	14.1	31.6
Zasavska	264	172.9	48.6	16.0	32.3
Spodnje- posavska	885	79.0	49.6	15.9	30.6
Jugovzhodna	2675	52.1	49.6	14.6	23.9
Osrednje- slovenska	2555	195.3	48.6	14.9	20.8
Gorenjska	2137	92.9	48.9	15.0	24.5
Notranjsko- kraska	1456	35.1	49.7	16.3	19.2
Goriska	2325	51.1	49.5	17.0	17.3
Obalno- kraska	1044	101.0	49.4	16.4	18.8

**Table 2:** Minimum and maximum suicide rates, socioeconomic and mental health service availability factors for 12 Slovenian statistical regions for the period from 2000 to 2009*

Parameter	Min.	Max.
Suicide rate	7.7	56.0
Marriage rate	2.3	4.2
Divorce rate	0.6	1.9
GDP	7928	25950
Unemployment rate	4.3	17.7
Antidepressants	80	294
Anxiolytics	176	412
AD/ANX	0.24	1.04
Alcohol use disorders	1	10
Psychotic disorders	2	11
Depression disorders	6	33
GP/FP rate	29.5	63.1
Clinical psychologist rate	0.0	7.5
Psychiatrist rate	1.2	8.8

*GDP – gross domestic product per capita; AD/ANX- antidepressant/anxiolytic ratio; GP/FP rate – number of general practitioners and family physicians per 100 000 inhabitants.

### Cross-correlation among suicide rate, socioeconomic, and mental health service availability factors

Marriage rate (r = -0.32, *P* < 0.001), divorce rate (r = -0.27, *P* < 0.001), GDP (r = -0.50, *P* < 0.001) antidepressant/anxyolitic ratio (r = -0.45, *P* < 0.001), and psychiatrist rate (r = -0.38, *P* < 0.001) were significantly negatively related to the suicide rate (Table 3). Unemployment rate (r = 0.65, *P* < 0.001), prevalence of identified alcohol use disorders (r = 0.35, *P* < 0.001), and prevalence of identified psychotic disorders (r = 0.25, *P* < 0.001) were significantly positively related to the suicide rate. Clinical psychologist rate, GP/FP rate, and the prevalence of identified depressive disorders were not significantly related to the suicide rate and were excluded from the subsequent GLMM. Since GDP was significantly related to the unemployment rate (r = -0.62, *P* < 0.001), it was excluded from the GLMM, too. Since marriage and divorce rate were significantly connected in a positive manner (r = 0.21, *P* = 0.021), the marriage/divorce ratio was included in the GLMM analysis.

**Table 3:** Pearson correlation coefficients between the suicide rate, socioeconomic factors, mental health service availability, and mental disorders factors (n = 120)*

Factor	Suicide rate	Marriage rate	Divorce rate	GDP	Unemployment rate	AD/ ANX	Alcohol use disorders	Psychotic disorders	Depression disorders	GP/FP rate	Clinical psychologist rate	Psychiatrist rate
Suicide rate	1.00	−0.32 ^†^	−0.27 ^†^	−0.50 ^†^	0.65 ^†^	−0.45 ^†^	0.35 ^†^	0.25 ^†^	0.09	−0.03	0.04	−0.38 ^†^
Marriage rate		1.00	0.21 *P* = 0.021	0.24 ^†^	−0.45 ^†^	0.06	0.12	0.17	0.21 *P* = 0.022	−0.11	−0.03	0.44 ^†^
Divorce rate			1.00	0.38 ^†^	−0.24 *P* = 0.012	0.13	−0.41 ^†^	−0.25 ^†^	0.07	−0.05	−0.01	0.44 ^†^
GDP				1.00	−0.62 ^†^	0.64 ^†^	−0.55 ^†^	−0.46 ^†^	−0.30 ^†^	0.06	0.36 ^†^	0.56 ^†^
Unemploy-ment rate					1.00	−0.54 ^†^	0.32 ^†^	0.17	0.00	−0.10	0.08	−0.31 ^†^
AD/ ANX						1.00	−0.43 ^†^	−0.35 ^†^	0.04	0.01	0.17	0.30 ^†^
Alcohol use disorders							1.00	0.90 ^†^	0.60 ^†^	−0.02	−0.00	−0.39 ^†^
Psychotic disorders								1.00	0.71 ^†^	−0.07	−0.04	−0.19
Depressive disorders									1.00	−0.18	−0.15	0.13
GP/ FP rate										1.00	−0.15	−0.03
Clinical psychologist rate											1.00	0.31 *P* = 0.005
Psychiatrist rate												1.00

*GDP – gross domestic product per capita; AD/ANX- antidepressant/anxiolytic ratio; GP/FP rate – number of general practitioners and family physicians per 100 000 inhabitants.

†*P* < 0.001.

### Factors related to the regional suicide rates

GLMM analysis showed that unemployment rate, marriage/divorce ratio, prevalence of alcohol use disorders per 1000 inhabitants, higher psychiatrist availability, and antidepressant/ anxiolytic ratio higher than 0.5 were significantly related to the regional suicide rates (Table 4). Among the socioeconomic factors, unemployment rate ranked as the most powerful predictor of suicide and an increase of one unit in the unemployment rate increased regional suicide rate by 2.21 (β = 2.21, 95% CI = 1.87- 2.54, *P* < 0.001). Marriage/divorce ratio was negatively related to the suicide rate and an increase of one unit in marriage/divorce ratio reduced regional suicide rate by 1.16 (β = -1.16, 95% CI = -2.20 to -0.13, *P* < 0.031). The most influential mental health service availability parameter was higher psychiatrist availability, which was negatively correlated with the suicide rate and reduced the regional suicide rate by 2.95 (β = -2.95, 95% CI = -4.60 to -1.31, *P* = 0.002). Another negatively correlated factor was antidepressant/anxiolytic ratio higher than 0.5, which reduced the regional suicide rate by 2.32 (β = -2.32, 95% CI = -3.75 to -0.89, *P* = 0.003). Among mental health disorders, only the prevalence of alcohol use disorders was significantly related to suicide rate and one unit increase in the prevalence of alcohol use disorders per 1000 inhabitants increased the regional suicide rate by 0.02 (β = 0.02, 95% CI = 0.01- 0.03, *P* = 0.008).

**Table 4:** General linear mixed model analysis (n = 120)

Factors	Regression coefficient (β)	t	*P*	95% confidence interval
Unemployment rate	2.21	15.64	<0.001	1.87- 2.54
Marriage/divorce ratio	-1.16	-2.51	0.03	-2.20 to -0.13
Prevalence of alcohol use disorders	0.02	3.47	0.008	0.01- 0.03
Higher psychiatrist availability	-2.95	-3.97	0.002	-4.60 to -1.31
Higher than 0.5 antidepressant/anxiolytic ratio	-2.32	-3.41	0.003	-3.75 to -0.89

Received: April 26, 2013

Accepted: October 15, 2013

## Discussion

The main finding of the present study was that besides socio-economic predictors, variations in suicide rates in different statistical regions in Slovenia may be influenced by unequal availability of mental health services and quality of depressive disorder treatment. It could be speculated that populations with high rates of socioeconomic deprivation have a higher need for mental health care, but the lowest access to it. The present results point to an uneven regional distribution of psychiatrists working at outpatient clinics and the most influential mental health service availability parameter in the present study was higher psychiatrist availability, which reduced the regional suicide rate by 2.95 and ranked as a protective factor against suicide. On the other hand, the availability of psychological services and the availability of primary care doctors did not have an impact on regional suicide rates. Kelleher (29) examined variations in suicide rates within Ireland and reported marked variation, with a 3-fold difference between the counties with the highest and the lowest rates, which indicates a need for improved services in areas of low population density and a high suicide rate (29).

Another important factor influencing the suicide rate in the present study was the antidepressant/anxiolytic ratio higher than 0.5, which reduced the regional suicide rate by 2.32. The ratio of anxiolytics to antidepressants has been described as a quality indicator regarding depression treatment, which is in most cases combined with anxiety and increased suicide risk (30). A number of studies have found a relationship between an increase in national antidepressant prescribing and declining suicide rates (31-41) and have shown that increased prescribing of antidepressants may indicate improved diagnosis and treatment of depression (31-41). Whereas epidemiological studies have shown an association of lower suicide rates with higher rates of antidepressant use, clinical trials of antidepressants in children and adolescents have shown an increased risk of suicidal thoughts or behavior in those who received antidepressants compared to those who received placebo (42,43). In contrast, other authors still contend that there is a bias in these findings and that the benefit is in fact greater than risk (43). A meta-analysis of 27 randomized controlled trials examined antidepressant prescribing in children and adolescents to age 18 with a diagnosis of major depressive disorder and showed that benefits far outweighed a small increased risk of suicidal behavior (44). Effective use of antidepressants requires administration to patients who have been properly diagnosed and adequately followed-up. On the other hand, studies that investigate the impact of prescription of anxyolitics on suicide rate are scarce (45-47). Sedatives and hypnotics are widely prescribed to elderly persons with symptoms of depression, anxiety, and sleep disturbance, although they have been associated with an increased risk for suicide in the elderly (47).

Some studies revealed that 80%-95% of suicide victims had a psychiatric illness and that the most common psychiatric disorder in suicide was untreated major depression (48-50). In the present study there was no association between the prevalence of depressive disorders and regional suicide rates. The absolute number of patients diagnosed with depressive disorders was low, ranging from 6 to 33 per 1000 population per year. This suggests that depressive disorders were underdiagnosed. In the present study, out of all the mental health disorders, only the prevalence of alcohol use disorders was weakly related to regional suicide rates. One unit increase in the prevalence of alcohol use disorders per 1000 inhabitants increased regional suicide rate by 0.02. Slovenia is among the European countries with the highest overall consumption of alcohol per capita. In the period 2000-2007, the per capita alcohol consumption was between 11.2 and 13.5 L of registered and 5 to 7 L of unregistered pure alcohol per adult inhabitant per year in Slovenia (51). Alcohol use was consistently implicated in the precipitation of suicidal behavior and individuals with alcohol use disorders were at an increased risk of suicide as compared to the general population (50,52,53). Particularly the prevalence of alcohol psychosis, was an important predictor of regional suicide rates in Slovenia (54).

In the present study the unemployment rate ranked as the most powerful socioeconomic predictor for suicide and an increase of one unit in the unemployment rate increased the regional suicide rate by 2.21. This finding is consistent with findings in other studies (55,56). Schneider (55) found that unemployment was associated with highly increased suicide risk, independently of the categorized psychiatric diagnosis. Ying (56) on the other hand reported that in terms of suicide risk unemployment affected men more than women. Another important socioeconomic parameter in our study, the higher marriage/divorce ratio was negatively associated with suicide rate and ranked as a protective factor. An increase of one unit in marriage/divorce ratio reduced the suicide rate by 1.16. Many studies have shown marriage to be protective against suicide (56-58). Masocco (58) concluded that for both men and women, being unmarried, widowed, or divorced/separated was associated with a higher suicide rate.

The present study has some limitations. Suicide may be an outcome of a number of different contributing factors (14). However, we focused on mental health service availability factors and their contributions to the regional suicide rates. Such limitations are present in all ecological studies in which there is no information available about the individual members of the compared populations. Additionally, in the present study we considered only the outpatient psychiatric service without including other psychiatric health service facilities. The number of psychiatrists was arbitrarily set at the level of 4 psychiatrists and more working at an outpatient clinic per 100 000 inhabitants and the antidepressant/anxiolytic ratio was arbitrary set to 0.5; however other arbitrary levels could be used in other populations. Finally, Slovenia is a small country with only 2 million inhabitants, so statistical analysis in our study was based on 12 statistical regions and further division into smaller regions would not produce a sufficient number of cases for statistical analysis. However, the whole population of Slovenia was included in the study.

In conclusion, to lower suicide rate in Slovenia we can focus on mental health service availability and mental disorders treatment. We suggest introduction of measures that would increase psychiatrist availability in statistical regions with socioeconomic deprivation and higher suicide rate.
